# Human Papillomavirus (HPV) Health Savings as an Alternative Solution: HPV Vaccination Behavior in Adolescents

**DOI:** 10.31557/APJCP.2021.22.2.471

**Published:** 2021-02

**Authors:** Wiwin Lismidiati, Ova Emilia, Widyawati Widyawati

**Affiliations:** 1 *Department of Pediatric and Maternity Nursing, Faculty of Medicine, Public Health and Nursing, Universitas Gadjah Mada, Yogyakarta, Indonesia. *; 2 *Department of Medical Education and Bioethics, Faculty of Medicine, Public Health and Nursing, Universitas Gadjah Mada, Yogyakarta, Indonesia. *

**Keywords:** Human papillomavirus, vaccination, savings, health education, adolescent

## Abstract

**Objective:**

This study aimed to measure the effects of reproductive health savings (tabungan kesehatan reproduksi, Takespro) on human papillomavirus (HPV) vaccine initiation program and the quality of the decision making to get vaccinated, as measured by knowledge, attitudes, beliefs, and self-efficacy toward HPV vaccination.

**Methods:**

This quasi-experimental study was conducted on 128 students randomly allocated to intervention and control groups. This research was conducted in junior high schools. The intervention group received the health education “Takespro HPV” intervention through videos and booklets for 6 months at school. Participants in the control group received usual care from the school. Data were collected using a questionnaire of knowledge, attitudes, beliefs, and self-efficacy about HPV vaccination modified by researchers based on previous research and tested for validity and reliability. Data were analyzed using Mann–Whitney test and independent t-test.

**Results:**

A total of 40 participants were actively enrolled in the intervention group, and 88 were passively enrolled in the control group. The health education that was part of the Takespro HPV intervention improved the knowledge (p < 0.05) and self-efficacy (p < 0.05) of the intervention group compared with the control group. The attitude and belief variables showed no significant difference (p > 0.05). Forty students exhibited the health reproduction savings behavior at schools. However, the savings amount was insufficient to get HPV vaccination at the initiation phase.

**Conclusion:**

“Takespro” HPV intervention can be considered an alternative to increasing the coverage of HPV vaccination in adolescents in Yogyakarta.

## Introduction

World Health Organization (WHO) recommends human papillomavirus (HPV) vaccination for girls alongside screening and treatment for older women to reduce cancer risks. HPV vaccination is most useful for girls aged 9–13 years and 14–26 years who have not been vaccinated before they are exposed to the virus (WHO, 2018). HPV is contagious within a few years through sexual contact and is usually obtained first after engaging in sexual activity (Wigle et al., 2013). The HPV vaccine can provide 89% protection (Cunningham, 2015). It effectively prevents cervical cancer with varying vaccination coverage.

Systematic reviews reported informational, behavioral, and environmental interventions to increase HPV vaccination. Informational intervention includes enhanced knowledge about HPV, cancer-related HPV related to cancer, or HPV vaccine. Behavioral interventions focus on individuals and healthcare providers, and environmental interventions include school policies or other policies, such as centralized and local governments’ involvement (Walling et al., 2016). School-based vaccination can also increase vaccination rates (Smulian et al., 2016). Furthermore, vaccination programs with insurance patterns reduce families’ costs (Smulian et al, 2016; Daley et al, 2014).

The HPV vaccination program in Indonesia is carried out in Jakarta and Yogyakarta. The central government collaborates on funding with the Global Alliance for Vaccine and Immunization and State Budget spending. In Bali, HPV vaccination is funded by the regional budget spending. Badung District provides free vaccinations from the regional budget fund of 17.8% (1567 from 8784) to public school students (Karnelli et al., 2013). The government cannot cover all the expenses of the vaccination. Therefore, an alternative strategy to solve this limitation is needed, such as individual funding vaccination.

The school environment is an essential target for providing the HPV vaccine to adolescents (Ricket et al., 2015). School-based information can raise awareness and knowledge about HPV prevention, increases prevention behaviors for sexually transmitted infections in general, and reduces sexual risks. In Indonesia, school-based vaccination programs through school children’s months may be an appropriate alternative to improving vaccination coverage (Arifah et al., 2017). The high vaccination coverage of countries that already have a school-based national is strong evidence that it can promote HPV vaccination (Kessles et al., 2012; Remes et al., 2014).

The prevention of cervical cancer by promoting HPV vaccination education at school has not yet been carried out in Indonesia. The HPV vaccination of adolescents is a new intervention to increase acceptance of the vaccine. Therefore, adolescents must be well-informed about the benefits and risks of vaccinating (Pelucchi et al., 2010). Parental approval is also essential. Adolescents generally assume that they are passive participants in determining medical decisions (Fernandez et al., 2014).

Financing of the HPV vaccine itself has not been budgeted into health security like the body that organizes social security. Vaccination requires considerable funding. Subsidies by the local government are not certain. Thus, community participation is needed for programs to be sustainable and can include more goals. For the sustainability of the HPV vaccination program in preventing cervical cancer, schools must be funded to buy vaccinations on their own and increase HPV vaccination in adolescents.

This study aimed to measure the effectiveness of Takespro HPV on the HPV vaccination rate among adolescents in Yogyakarta. Takespro is an intervention in promoting adolescents’ vaccination behavior, including education to adolescents, parental empowerment, and reproductive health savings. In this research, the intervention consists of health education about HPV and the HPV vaccine for adolescents and their parents, information to their parents, and the reproduction health savings collected for 6 months. The savings funds will be used to initiate the first dose of HPV vaccination. The factors affecting HPV vaccination behavior were also measured to determine whether health education will improve knowledge, attitudes, beliefs, and self-efficacy to HPV vaccination.

## Materials and Methods

*Study design*

The design of this research was a quasi-experimental with non-equivalent (pretest and post-test) control group design. The study was conducted for 6 months, from October 2018 to April 2019. The effectiveness of intervention was known by comparing knowledge, attitudes, beliefs, and self-efficacy and the effects of savings behavior on student health. Trial registration was not needed to conduct this study.

*Ethical Approval*

This study was approved by the Ethics Commission of the Faculty of Medicine and Health with the number KE/FK/1100/EC/2017.

*Sample and recruitment*

This study enrolled students in the first high schools in the Special Region of Yogyakarta. Among other considerations for selection, this school has the Adolescent Reproductive Health Counseling Program. First secondary schools in Bantul and secondary schools in Sleman were selected because of their characteristics. Seventh- and eighth-grade students who have not had an HPV vaccination and are willing to be respondents and follow the entire research series were included in this study. Students who are unwilling to save up at school were excluded.

The sample size calculation was established based on the sample’s formula size to test the one-sided hypothesis on the different means from two populations. With α 95% and β 90%, the minimum number of samples was 42 respondents. In anticipation of samples that drop out, 10% was added to a total sample minimum of 46 respondents per group.

*Research Instrument*

This study used five instruments to measure the research variables: 1) knowledge questionnaires of demographic characteristics, knowledge questionnaires made by researchers themselves considering several similar studies (Strohl et al., 2015; Khan et al., 2016), 2) modified behavior questionnaires by researchers considering the Carolina HPV Immunization Attitudes and Beliefs (2009) questionnaires (Donadiki et al., 2014; Christine et al., 2013), 3) the belief questionnaire using HBM theories (Karneli et al., 2013; Ricket et al., 2014; Donadiki et al., 2014; Christine et al., 2013), 4) a self-efficacy questionnaire modified by self-efficacy-related research for pap smear screening (Hogenmiller et al., 2007), and 5) the HPV vaccination behavior questionnaire (Gerend et al., 2012). Eight experts in maternity nursing/women reproductive and 1 Ph.D. in nurse oncology for content validity. The questionnaire was tested on 27 students in junior high school for clarity and ease of reading and 99 students for construct validity. Revisions were made based on content expert and pilot subjects’ recommendations. 

*Knowledge of HPV and HPV Vaccine*

Twenty-three multiple-choice questions assess the knowledge of HPV, cervical cancer, and HPV vaccine. Correct responses in this section were added up. A higher mark indicates better knowledge, with score of 23 as the highest. 

*Attitudes toward HPV Vaccine*

Attitude towards HPV, cervical cancer, and the HPV vaccine (15 items). The items are on a 5-point Likert scale from 1 “strongly disagree” to 5 “strongly agree”.

*Beliefs toward HPV Vaccine*

Sixteen items evaluate 4 areas of health belief: efficacy of vaccine (4 items), cost and availability (5 items), adverse effects of vaccine (6 items), and recommendations from others (3 items). The items are on a 5-point Likert scale from 1 “strongly disagree” to 5 “strongly agree”.

*Self-Efficacy toward HPV Vaccine*

Self-efficacy towards HPV, cervical cancer, and the HPV vaccine (22 items). The items are in a 5-point Likert scale from 1 “strongly disagree” to 5 “strongly agree”; 

Overall, the scale-level content validity index of the questionnaire was found to be more than 0.6, which is an acceptable level.

*Reproductive Health Savings*

The health saving was introduced to enable the purchasing power of students and parents of the HPV vaccine to uptake HPV vaccination behavior.

The deposit of health savings:

1). The students who had signed up and obtained a health-saving book made direct deposits to the teacher that the researchers had appointed, 2). The amount of savings that students put up is determined by their parents’ economic capability, 3). All savings were deposited in the bank using different schools’ accounts. 

*Data collection*

The pretest was conducted in both groups. A questionnaire was used to measure knowledge, attitudes, beliefs, and self-efficacy on HPV vaccination behavior prior to the intervention.

Researchers and school teachers in the intervention group promoted the program on health education and reproductive health savings. Eligible respondents in the intervention group received health education through videos on HPV and HPV vaccine prepared by obstetricians and gynecologists. They were also encouraged to have an account for their reproductive health savings. All participants in the control group received regular health education from schools. After 4 weeks, all respondents in both groups were asked to fill out a post-test questionnaire. During 6 months of the savings period, each student’s savings were calculated to finance the first dose (initiation dose) of HPV vaccination. The girls in the control group were also observed for 6 months whether they were vaccinated at school based on teachers’ information.

*Statistical analysis*

Descriptive statistics for continuous variables were expressed as mean or median. Categorical variables were presented as frequency and proportion. All statistical tests were two-sided. The chi-square test and Fisher’s exact test were used to determine the two groups’ homogeneity test . Different scores for knowledge, attitude, belief, and self-efficacy were analyzed using the Mann–Whitney test and independent t-test. Multivariate logistic regression analysis was performed to determine the disparity between the two groups, such as the history of hearing about HPV and sources of information once obtained through the Internet.

## Results

A total of 138 female students participated in this study. Forty respondents were enrolled in the intervention group, and 88 were enrolled in the control group. Ten people at the control group dropped out and did not show up in the post-test.

The baseline demographic characteristic of the study subjects were shown in Table 1. The mean age was 13.3 ± 0.82. The majority of them were Moslem (95.3%) followed by Others (4.7%) and living with parents (96.9%) followed by other (3.1%). 

Upon analysis, the intervention and control group were well matched, and there was no statistically significant difference between these two groups except in have heard about HPV and got information about HPV from the internet characteristics. The regression test result showed that only educational intervention had a significant value p < 0.05 (Table 2).

*Knowledge for HPV and HPV vaccination, Attitude, Beliefs and Self-efficacy about HPV vaccination*

Both intervention and control groups demonstrated knowledge with the median score of min-max (-3-11) and (-9-7). There were statistically significant differences between the two groups (p=0.016); Otherwise, the attitude with the mean score of 3 ± 4.7 and -2 ± 3.5. There were no statistically significant differences in the total mean scores between the two groups (p=0.576).

The mean score of beliefs toward the HPV vaccine was 1.8 ± 4.0 and 0.6 ± 3.4 respectively. There were no statistically significant differences in the total mean scores between the two groups (p=0.110). Otherwise, the mean score of the self-efficacy was 3.7± 7.9 and 0.6± 4.3 respectively. There were statistically significant differences in the total mean scores between the two groups (p=0.110).

Differences in knowledge scores and self-efficacy were found between the intervention and control groups (Table 3).

*Health Savings*

Reproductive health savings as a guarantee in financing HPV vaccination independently are carried out on 40 students in the intervention group who save at school for six months from November 2018 to April 2019.

Based on Table 4, it has been found that the amount of student savings in the intervention group is at least IDR 63,000 and IDR 200,000 collected over a period of 6 months. The results of health savings at this school may be illustrated that those who have not been able to get HPV vaccination by using reproductive health savings.

The results of reproductive health savings on the intervention group showed that the time limit for saving students in this study was so short that little money was collected (Table 4).

**Table 1:** Baseline Characteristics

	Intervention (n = 40)		Control (n = 88)		p
	N	%	N	%	
Age (years)	13.3 ± 0.82		13.33 ± 0.50		0.330c
Religion					
Muslim	40	100	82	93.2	0.081c
Christian	0	0	2	2.3	
Catholic	0	0	4	4.5	
Health Insurance					
Yes	33	82.5	65	73.9	
No	7	17.5	23	26.1	0.285a
Source of information Teacher					
Yes	16	40	37	42	0.828a
No	24	60	51	58	
Internet					
Yes	5	12.5	33	37.5	0.004a
No	35	87.5	55	62.5	
Have heard about HPV					
Yes	13	32.5	60	68.2	0.000a
No	27	67.5	28	31.8	
Living with parents					
Yes	37	92.5	87	98.9	0.090b
No	3	7.5	1	1	

a, chi-square test; b, Fisher’s exact test; c, Mann–Whitney test

**Table 2:** Regression Test for the Characteristic of the Respondents with Educational Interventions on the Intervention and Control Groups

Model	Understandarized Coefficient		Standarized Coefficient	t	Sig	95% CI for ᵝ	
	β	SE	Beta			Lower Bound	Upper Bound
Educational Intervention	2.054	0.678	0.286	3.032	0.03	0.713	3.395
Sources of Information : internet	0.328	0.652	0.045	0.502	0.616	-0.963	1.169
Have heard about HPV	0.782	0.618	0.116	1.266	0.2	-0.44	2.005

*, logistic regression

**Table 3:** Differences in Discrepancies Pretest and Post-Test to Knowledge, Attitudes, Beliefs, and Self-Efficacy

	Intervention (n = 40)	Control (n = 88)	p value
Δ Knowledge	2 (-3–11)	1 (-9–7)	0.016a
Δ Attitude	3 ± 4.7	-2 ± 3.5	0.576b
Δ Belief	1.8 ± 4.0	0.6 ± 3.4	0.110b
Δ Self Efficacy	3.7± 7.9	0.6± 4.3	0.022b

Mann-Whitney test^a^, Independent t-test^b^

**Table 4:** Saving Behavior of the Students for 6 Months in the Intervention Group (n = 40)

Saving	N (%)	Total savings (in IDR)
<100,000	22 (55)	63,000–200,000
>100,000	18 (45)	

## Discussion

Our intervention is built on an understanding of how a health savings model can affect health behavior. The study suggests that health savings based on economic principles that are part of “Takespro HPV” intervention are sufficiently effective in promoting HPV vaccine initiation and early dose resolution in adolescents.

This study was also designed to measure the knowledge, attitude, belief, and self-efficacy that can affect decision making about HPV vaccination. The intervention group received interventions, including education on HPV and the HPV vaccine, empowerment of parents, and reproductive health savings. In addition to health education, the target of saving money is to alleviate vaccination financing.

Kim et al., (2019) found that most respondents receiving cervical cancer prevention education had significant improvement in adolescents’ cervical cancer understanding. Chinwe et al., (2015) also found that their respondents had relatively good knowledge of cervical cancer and cervical cancer screening after receiving health education. Knowledge acquired from health education can positively affect individual behavior on cervical cancer screening due to the correctness of the information, and thus the respondents received the message delivered.

The health promotion’s success may be influenced by several factors, such as models, methods, material, sources, and media used. The models, methods, and media used in delivering health information must be adjusted to the target to achieve optimal results. In this study, media exposure using videos was conducted by the researcher twice, and the booklets were distributed to the respondents. In making the booklets and this video as the media education, researchers employed the informative approach to use the facts related to HPV and cervical cancer to present clear, enlightened images. The argumentative approach was established in logical arguments for adolescents and parents with their language.

Students who received education about HPV and the HPV vaccine demonstrated improved knowledge and enhanced strategy. The acquired knowledge changed one’s perspective on judging things. This result is in accordance with the research conducted by Ratnasari (2015), which mentioned that knowledge could affect behavior. The result is consistent with the view that a higher level of knowledge corresponds to a better level of detachment. If a person has a lower knowledge, the level of self-promotion of an object is included in this matter, absorbing health information to prevent cervical cancer by HPV vaccination. Rachmani et al., (2012) found a relationship between the level of knowledge and a person’s self-efficacy.

In the present study, the amount of savings was insufficient to initiate HPV vaccination. The students’ ability to save money was determined by the amount of the student’s pocket money and the financial ability of the family and the saving period. Studies have shown that some parents have jobs as self-employed and as housewives. Six months of savings is insufficient to initiate the HPV vaccination at school. A part of the cost of coed teenage vaccination factor still depends on the parents’ decision. 

Adolescents with sufficient knowledge about vaccination and are willing to receive HPV vaccination still require parental consent. Studies show that most parents approve when they understand the risks and benefits of vaccination. Another factor considered is the recommendations of the Indonesian Pediatric Association and the effectiveness of the HPV vaccine. Some previous studies have concluded that the denial of vaccination is caused by the perception that vaccines are unnecessary because they are without risk, without the recommendation of doctors or other medical personnel to vaccinate, worried about the safety and side effects of vaccines, and insufficient knowledge of the vaccine (Blackman et al., 2013; Kester et al., 2013).

Another obstacle to the implementation of health savings in this study is the promotion of health savings to less-optimum students and parents. Success in health education is also affected by some factors involved in media coverage. Media coverage is a major factor affecting the personal and parental beliefs about HPV vaccines (Redman, 2013). Coverage of media exposure and the distortions of messages in the media affect five basic beliefs, including perceptions of vulnerability, obstacles, severity, norms, and self-efficacy, influencing vaccine decision-making. The message’s contents of the incremental approach in media education developed in this study are not yet so powerful in framing media messages.

Free vaccine and vaccination incorporated into government programs belong to things considered by adolescents and parents. The role of government through policy is to make vaccines freely available and accessible to reduce cost problems. Countries that deliver HPV vaccinations for free have higher vaccination rates. HPV vaccination programs with school-child immunization programs in Indonesia may be a viable solution to increase vaccination coverage.

The high vaccination coverage of countries that already have a school-based national HPV vaccination program strongly suggests that the program effectively increases HPV vaccination coverage. Students’ desire to save for their reproductive health in this regard contributes to their positive behavior toward HPV vaccination. In addition, a school program for reproductive health savings for students can be a prototype of an intervention to promote HPV vaccination behavior. Before promoting vaccines and education to teachers, parents and adolescents are needed to increase awareness and willingness in receiving the HPV vaccine. This study’s limitations are the message compound in the education media and the information bias of respondents.

In conclusion, we can mention that the government has to increase vaccination coverage by initiating a national school-based HPV vaccination program. This program’s implementation includes community education where education can be given through health care, teachers at schools, and socially through electronic media. Further research should determine savings in reproductive health at private schools and the parents’ willingness to vaccinate adolescents.

## References

[B1] Arifah K, Damayanti W, Sitaresmi MN (2017). Human papillomavirus acceptability among female adolescent in Yogyakarta. Sari Pediatri.

[B2] Blackman E, Thurman N, Halliday DAC (2013). Multicenter study of human papillomavirus and the human papillomavirus vaccine: Knowledge and attitudes among people of African Descent. Infect Dis Obstet Gynecol.

[B3] Cunningham MS, Skrastin E, Fitzpatrick R (2015). Cervical cancer screening and HPV vaccine acceptability among rural and urban women in Kilimanjaro Region, Tanzania. BMJ Open.

[B4] Christine A, Putra AE (2013). Penerimaan vaksinasi kanker serviks pada siswi SMA di kabupaten Badung. Commun Health.

[B5] Chinwe ER, Abigail UR (2015). Impact of health education on knowledge, attitude and practice of cervical cancer screening among secondary school teachers in Enugu State. J Womens Heal Care.

[B6] Daley MF, Kempe A, Pyrzanowsky J (2014). School-located vaccination of adolescents with insurance billing: Cost, reimbursement, and vaccination outcomes. J Adolesc Health.

[B7] Donadiki EM, Garcia RJ, Barrera VH (2014). Health belief model applied to non-compliance with HPV vaccine among female university students. Public Health.

[B8] Fernández ME, Le YCL, Espada NF (2014). Knowledge, attitudes, and beliefs about human papillomavirus (HPV) vaccination among Puerto Rican mothers and Daughters : A qualitative study. Prev Chronic Dis.

[B9] Gerend MA, Shepherd JE (2012). Predicting human papillomavirus vaccine uptake in young adult women: Comparing the health belief model and theory of planned behavior. Ann Behav Med.

[B10] Hogenmiller JR, Atwood JR. Lindsay AM (2007). Self-efficacy scale for pap smear screening participation in sheltered women. Nurs Res.

[B11] Harper DM, Verdenius I, Harris GD (2014). The influence of free quadrivalent human papillomavirus vaccine (HPV4) on the timely completion of the three dose series. Prev Med.

[B12] Karnelli NK, Suwiyoga K, Sudibya A (2013). Parental willingness to pay the cervical cancer vaccination cost of senior high school aged students in Badung Regency. J Prev Med Public Health.

[B13] Khan TM, Buksh, MA, Rehman EU (2016). Knowledge, attitudes and perception towards human papillomavirus among university students in Pakistan. Papillomavirus Res.

[B14] Kessels SJM, Marshall HS, Watson M (2012). Factors associated with HPV vaccine uptake in teenage girls: A systematic review. Vaccine.

[B15] Kim HW, Lee YJ, Lee DB (2019). Effects of cervical cancer prevention education in middle-school girls in Korea: A mixed-method study. Heliyon.

[B16] Kester LM, Zimet GD, Fortenberry JD et al (2013). A national study of HPV vaccination of adolescent girls: Rates, predictors, and reasons for Non-Vaccination. Matern Child Health J.

[B17] McRee AL, Brewer NT, Reiter, PL (2010). The Carolina HPV immunization attitudes and beliefs scale (CHIAS): Scale development and associations with intentions to vaccinate. Sex Transm Dis.

[B18] Pelucchi C, Esposito S, Galeone C (2010). Knowledge of human papillomavirus infection and its prevention among adolescents and parents in the greater Milan area, Northern Italy. BMC Public Health.

[B19] Ricket VI, Auslander BA, Cox DS (2014). School-based vaccination of young US males: Impact of health beliefs on intent and first dose acceptance. Vaccine.

[B20] Remes O, Smith LM, Llano BEA (2014). Individual- and Regional-level determinants of Human papillomavirus (HPV) vaccine refusal : The Ontario Grade 8 HPV vaccine cohort study. BMC Public Health.

[B21] Ratnasari D, Kartika RD (2015). Hubungan antara pengetahuan mengenai kanker serviks terhadap keikutsertaan pada program deteksi dini kanker serviks di Kecamatan Cilongok Kabupaten Banyumas. Sainteks.

[B22] Rachmani B, Shaluhiyah Z, Cahyo K (2012). Sikap remaja perempuan terhadap pencegahan kanker serviks melalui vaksinasi HPV di kota Semarang. Media Kesehatan Masyarakat Indonesia.

[B23] Redman S (2013). Media influence on human papillomavirus vaccine decision-making behavior. indigo.uic.edu. Accessed on April.

[B24] Strohl AE, Mendoza G, Ghant MS (2015). Barrier to prevention: Knowledge of HPV, cervical cancer and HPV vaccinations among African American women. Am J Obstet Gynecol.

[B25] Smulian EA, Mitchell KR, Stokley S (2016). Intervention to increase HPV vaccination coverage: A systematic review. Hum Vaccin Immunother.

[B26] WHO (2018). Protecting Adolescent with HPV vaccine.

[B27] Wigle J, Coast E, Jones DW (2013). Human papillomavirus (HPV) vaccine implementation in low and middle-income countries (LMICs): Health system experiences and prospects. Vaccine.

[B28] Walling EB, Benzoni N, Dornfeld J (2016). Interventions to improve HPV vaccine uptake: A systematic review. Pediatrics.

